# Hands-On or Gloved Approach? Unveiling Patient Experiences in Orthopaedic Foot and Ankle Clinics

**DOI:** 10.1177/19386400251333477

**Published:** 2025-05-08

**Authors:** Andrea Rincon, Yvonne Conway, Samuel K. Simister, Frank Sierra, Shannon Tse, Christopher Kreulen, Eric Giza

**Affiliations:** School of Medicine, University of California Davis, Sacramento, California; Department of Orthopaedic Surgery, University of California Davis, Sacramento, California; Department of Orthopaedic Surgery, University of California Davis, Sacramento, California; School of Medicine, University of California Davis, Sacramento, California; Department of Orthopaedic Surgery, University of California Davis, Sacramento, California; Department of Orthopaedic Surgery, University of California Davis, Sacramento, California; Department of Orthopaedic Surgery, University of California Davis, Sacramento, California

**Keywords:** outcome studies, statistical analysis, patient satisfaction, gloves, patient perspective, patient-centered care, medical examination gloves, physician-patient relation, quality improvement, patient experience

## Abstract

**Background:**

Literature suggests that patients view medical examination gloves as often overused and opportunities for cross-contamination. Little research focuses on the patient’s perception of their surgeon when performing a physical examination in a clinic with or without medical examination gloves. The purpose of this project was to determine patient preference for glove use during foot and ankle examinations.

**Methods:**

A cross-sectional survey was conducted in a Foot and Ankle Orthopaedic Clinic, with 99 out of 100 patients participating (99% response rate). Participants, 53 patient’s female (59.5%) and 36 male (40.4%) answered 8 questions on glove utilization, scored on a Likert scale (Appendix A). Descriptive statistics, data normalcy, and significance tests were used to analyze glove utilization (yes/no), gender, and age group, at P < .05 significance. The primary outcome examined patient preference for glove use during exams, while secondary outcomes assessed perceived thoroughness and provider approachability.

**Results:**

Among 89 patients (59.5% female, 40.4% male, mean age ~55), glove use was reported in 75 surveys and was more common with male patients (33.3% vs 9.43%, P = .059). Males were slightly more agreeable to preferring no gloves (2.86 vs 3.17, P = .134). Patients with physicians who wore gloves vs those without gloves during the encounter were more congenial to glove use for themselves (2.50 vs 3.24, P = .001) and for others (2.21 vs 3.00, P< .001), respectively. Patients preferred glove use for wound exams. Gender, glove use, or age did not influence perceptions of examination thoroughness (1.61) or provider approachability (3.32).

**Conclusion:**

Glove use did not negatively impact patient perception of physical examination thoroughness or personability onto their physician. The results suggest that glove use may be up to the provider’s discretion without concern for seeming less personable to patients or affecting patient perception of examination thoroughness.

**Levels of Evidence:**

Level IV

“The purpose of this project was to determine patient preference for glove use during foot and ankle examinations.”

## Background

The importance of sterility is emphasized early in a surgeon’s career. It is largely accepted that maintaining sterility in the operating room significantly reduces infection rates and provides safety advantages for all team members. Using gloves becomes second nature when examining a patient in and out of the operating room. While the necessity of glove use in the operating room is well-established, the patient’s perspective on glove use in outpatient settings is underrepresented in current research. According to the Centers for Disease Control and World Health Organization, the concept of universal precautions states that providers should wear medical examination gloves when the examination of a patient involves contact with blood, bodily fluids, mucous membranes, or non-intact skin.^1,2^ However, there is currently no standard protocol for wearing gloves when examining patients with intact skin.

Literature suggests that patients often perceive medical examination gloves as being overused or misused. In addition, patients have expressed concerns about the potential for cross-contamination with glove use.^3^ Minimal research has focused on patients’ perceptions of their orthopaedic surgeons’ use of medical examination gloves during clinic examinations. Through this study, we hope to identify patient perceptions and feelings regarding whether their orthopaedic surgeon chooses to wear medical examination gloves during a foot and ankle physical examination.

The aim of this study is to assess patient preferences regarding orthopaedic surgeons’ use of medical examination gloves during foot and ankle examinations in an outpatient clinic. By gathering this information, we aim to improve the patient experience during clinic visits and make more informed decisions regarding personal protective equipment use. We hypothesize that direct physician contact confers greater personability and examination thoroughness, and that patients may prefer their surgeon to wear gloves during a foot and ankle examination.

## Methods

Following Institutional Review Board Approval, a cross-sectional institutional study was conducted during a university Foot and Ankle Orthopaedic Subspecialty Clinic visit for patients willing to participate in the study from January 2024 to April 2024. Inclusion criteria encompassed all adult patients aged 18 and over who were seen in person in the foot and ankle clinic. All surgeons used gloves when examining an open or bleeding wound; no gloves were used with healed wounds or those without incisions. The medical examination gloves used in this study are Medline, SensiCareSilk with Smart Guard Nitrile gloves, which are contracted with the university clinic apart from this present study. In addition, all surgeons followed standard handwashing and hygiene protocols before and after every interaction with a patient. At the start of the survey, we collected basic demographic data such as patient age, gender, and reason for visit (eg, establishing care, pre-operative, or post-operative) (Flow Chart 1).

**Flow Chart 1.**
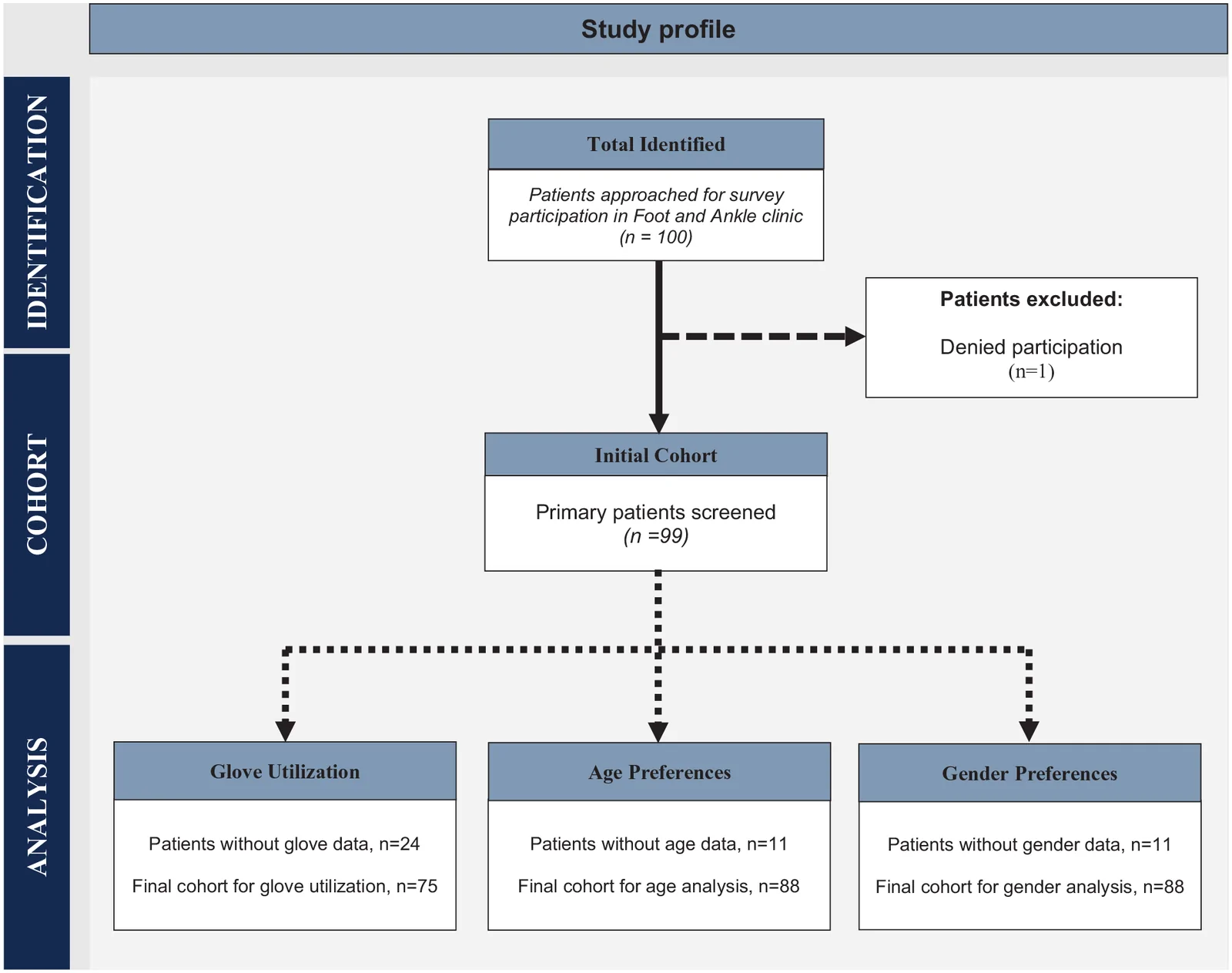
Study profile. Population numbers based on inclusion and exclusion criteria.

The survey development process followed a systematic approach that included a literature review, item selection, and questionnaire structure. To ensure relevance, the survey was supported by existing literature on patients’ preference for medical examination glove use and hand-hygiene practices.^2-4^ Literature suggests a few key themes such as patient comfort, personability and trustworthiness in providers, and infection control, which were all considered when crafting the survey. A team of surgeons and researchers collaborated to select key questions that were both relevant and straightforward, including ensuring that the questions were appropriate, relevant, and aligned to our study’s main purpose. We aimed to avoid ambiguity and unclear wording to ensure the questions were straightforward to answer.

After glove utilization reason for appointment, 5 questions were selected to identify patient preferences, including specific aspects of patient preference on provider glove use, examination thoroughness, personability, and trustworthiness toward the provider (Appendix A). Questions aimed to highlight patient perceptions on how glove use hinders or enhances the medical examination and assessed patients’ perceptions of whether they felt that their orthopaedic surgeon is friendly/warm, performed a thorough assessment, and whether they believe surgeons should always wear gloves during the examination. Answers for these questions were scored on a Likert scale, with 1 = strongly agree, 3 = neutral, and 5 = strongly disagree. Each survey was collected after the physical examination and clinic visit.

Descriptive statistics (mean ± standard deviation) were performed to analyze patient demographics, patient preference for glove utilization, and overall survey scores. For comparison between the 2 independent variables (glove use ± no glove use), an independent *t*-test was used to analyze the difference in means. For comparison, across the age groups, a 1-way analysis of variance (ANOVA) was used. Potential confounders such as age, gender, and visit type were considered during analysis, and we acknowledged that other factors such as previous health experiences, health status, educational status, and cultural differences were not included in the analysis. A *P*-value of <.05 was deemed significant. All statistical analyses were performed using SPSS (IBM, Chicago, Illinois).

## Results

The study invited 100 patients, with 99 of them being agreeable to participating in the survey, resulting in a response rate of 99%. For basic demographics, 53 patients (59.5%) identified as female and 36 (40.4%) identified as male. A total of 16 (16.1%) patients reported being under the ages of 39, 13 (13.1%) patients reported being between the ages of 40 and 49, 19 (19.1%) patients reported being between the ages of 50 and 59, 19 (19.1%) patients reported being between the ages of 60 and 69, and 21 (21.2%) patients reported being greater than 70 years old. Clinic visit types included 32 (32.3%) new patient consultations, 17 (17.1%) pre-operative appointments, 24 (24.2%) post-operative visits, and 16 (16.1%) follow-ups.

Patient preferences based on physician glove use during their physical examination totals based on age, gender, and reason for visit are listed in Table 1. In the 20 encounters where gloves were used by the surgeon, patients were slightly more agreeable to gloves being worn during their examination compared to patients who were examined without gloves (2.5 ± 0.827 vs 3.24 ± 0.823, *P =* .001) (Figure 1). Similarly, patients demonstrated a preference for their providers to wear gloves when examining other patients, while those examined without gloves remained neutral (2.21 ± 0.713 vs 3.00 ± 0.588, *P* < .001) (Figure 2).

**Table 1.** Comparison of Scores by Glove Use.

Overall	Gloves used	Gloves not used	*P*
Number of patient encounters, % (n)	32 (17/75)	68 (51/75)	
Female, % (n)	9.43 (5/53)	90.6 (48/53)	.059
Male, % (n)	33.3 (12/36)	44.4 (24/36)	
Age, mean ± SD	52.1 ± 20.7	54.1 ± 15.2	.671
Age group, % (n)			
<39	29.4 (5/17)	16 (8/50)	.850
40-49	5.88 (1/17)	18 (9/50)	
50-59	17.6 (3/17)	26 (13/50)	
60-69	23.5 (4/17)	20 (10/50)	
>70	23.5 (4/17)	20 (10/50)	
Reason for visit, % (n)			
New patient	40 (8/20)	36.4 (20/55)	.983
Pre-op	20 (4/20)	18.2 (10/55)	
Post-op	20 (4/20)	25.5 (14/55)	
Follow-up	20 (4/20)	20 (11/55)	

SD, standard deviation; PE, physical examination.

**Figure 1.**
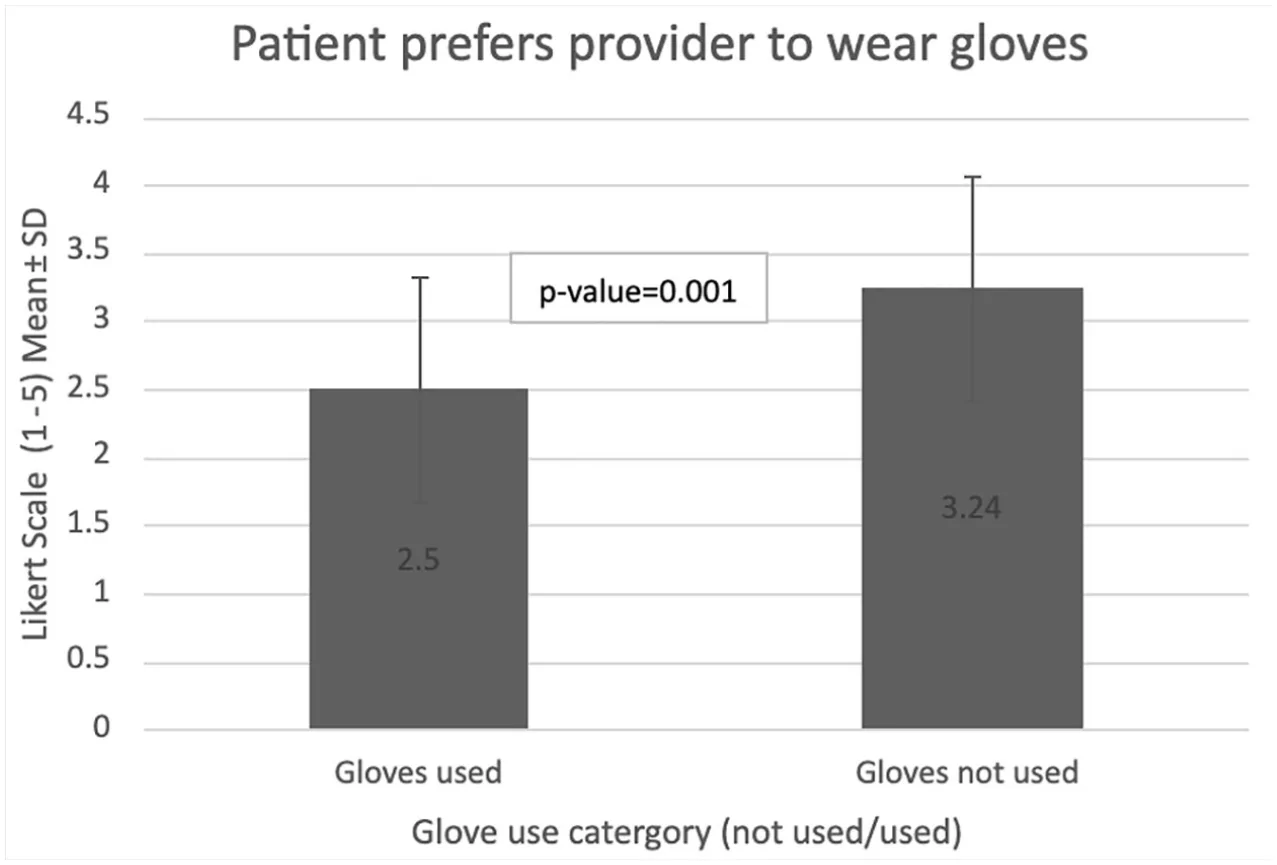
Mean and SD, standard deviation from survey response to “patient prefers provider to wear gloves” scored on a Likert scale (1-5) “1 = strongly agree, 2 = agree, 3 = no preference, 4 = disagree, 5 = strongly disagree” for the group that had gloves used or not used during the encounter.

**Figure 2.**
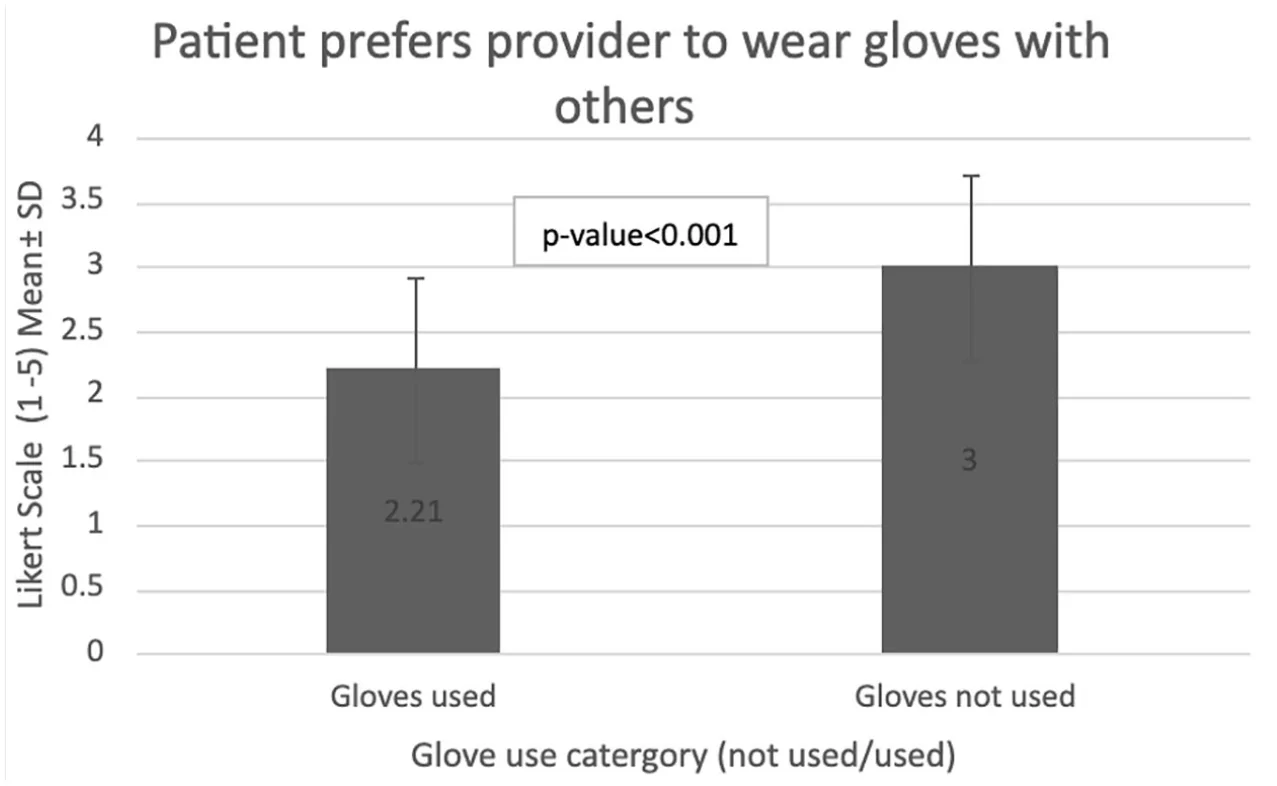
Mean and SD, standard deviation from survey response to “patient prefers provider to wear gloves with others” scored on a Likert scale (1-5) “1 = strongly agree, 2 = agree, 3 = no preference, 4 = disagree, 5 = strongly disagree” for the group that had gloves used or not used during the encounter.

It was found that patients examined by a physician with gloves slightly disagreed with the idea, or preference of, physicians not wearing gloves during a physical examination (3.35 ± 0.875), compared to those examined without gloves who remained relatively neutral (2.98 ± 0.858, *P =* .039) (Figure 3). Both groups agreed to prefer their physician wear gloves when examining a wound (2.16 ± 0.958 vs 2.17 ± 1.023). Wearing gloves had no effect on patient perceptions of the thoroughness of their examination (1.47 ± 0.612 vs 1.62 ± 0.745, *P =* .370). Neither group agreed with the idea that gloves made their physician encounter feel less personable (3.33 ± 0.686 vs 3.33 ± 0.952, *P =* .777).

**Figure 3.**
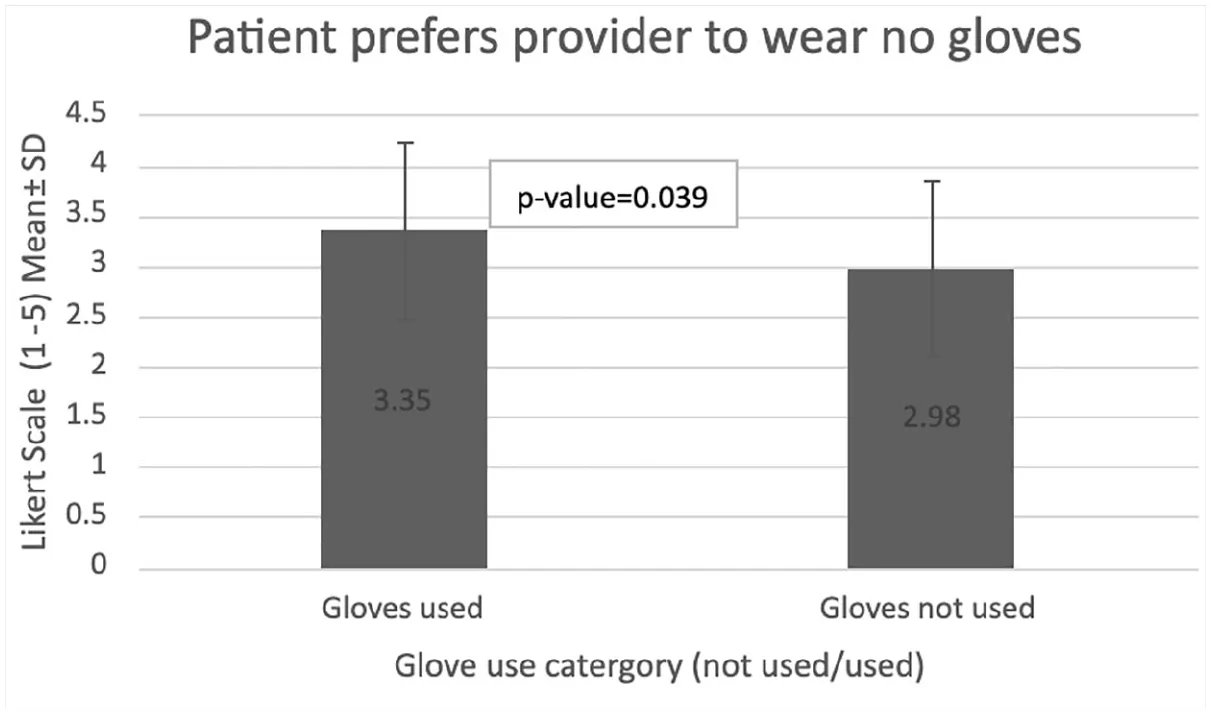
Mean and SD, standard deviation from survey response to “patient prefers provider to wear no gloves” scored on a Likert scale (1-5) “1 = strongly agree, 2 = agree, 3 = no preference, 4 = disagree, 5 = strongly disagree” for the group that had gloves used or not used during the encounter.

Regardless of gender, patients overall agreed that they received a thorough physical examination during their visit (1.61 ± 0.726). Both genders remained relatively neutral with the idea that gloves conferred less personability onto their providers (3.32 ± 0.874). In addition, both genders agreed to prefer glove use by their provider when wounds are being examined (2.19 ± 1.003). In an age group comparison, no significant differences were found among survey responses (Table 2). Survey questionnaire Likert-scale score comparisons by gender (Table 3) demonstrated no statistically significant differences in glove preferences or perception by those who self-identified as male or female.

**Table 2.** Comparison of scores by age groups.

	<39	40-49	50-59	60-69	>70	*P*
Total, % (n)^a^	18.1 (16)	14.7 (13)	21.5 (19)	21.5 (19)	23.8 (21)	
Survey questions, mean ± SD^b^						
Thorough PE?	2.0 ± 0.535	1.38 ± 0.650	1.67 ± 1.03	1.5 ± 0.516	1.47 ± 0.612	.241
Prefer gloves worn?	3.06 ± 0.854	3.69 ± 1.03	3.16 ± 0.898	3.00 ± 0.667	2.8 ± 0.768	.068
Prefer gloves worn with others?	2.75 ± 0.775	3.31 ± 0.855	2.84 ± 0.898	2.89 ± 0.567	2.78 ± 0.548	.332
Prefer NO gloves	2.87 ± 0.806	2.54 ± 1.05	3.21 ± 0.855	3.32 ± 0.820	3.05 ± 1.18	.252
Gloves feel less personable	2.93 ± 0.799	3.15 ± 1.07	3.53 ± 0.772	3.28 ± 0.895	3.56 ± 0.922	.320
Gloves with examining a wound	2.47 ± 1.13	2.23 ± 1.09	2.47 ± 1.26	1.94 ± 0.725	1.74 ± 0.733	.132

SD, standard deviation; PE, physical examination.

a 11 patients did not list age.

b Scores ranging 1 to 5, with 1 = strongly agree and 5 = strongly disagree

**Table 3.** Comparison of scores by gender.

	Overall	Female^b^	Male^b^	*P*
Total, % (n)	-	59.4 (53)	40.4 (36)	-
Survey questions,^a^ mean ± SD				
Thorough PE?	1.61 ± 0.726	1.67 ± 0.747	1.55 ± 0.711	.440
Prefer gloves worn?	3.08 ± 0.833	3.06 ± 0.842	3.14 ± 0.912	.650
Prefer gloves worn with others?	2.89 ± 0.709	2.87 ± 0.658	2.91 ± 0.866	.779
Prefer no gloves	3.03 ± 0.929	3.17 ± 0.923	2.86 ± 1.004	.134
Gloves feel less personable	3.32 ± 0.874	3.33 ± 1.013	3.33 ± 0.736	1
Gloves with examining a wound	2.19 ± 1.003	2.06 ± 0.958	2.3 ± 1.104	.281

SD, standard deviation; PE, physical examination.

a Scores ranging 1 to 5, with 1 = strongly agree and 5 = strongly disagree.

b Scores ranging 1-5, 1=strong agree, 5=strongly disagree.

## Missing Data

A total of 10 patients did not self-report their gender identity and were therefore excluded from gender analyses in the study. This exclusion ensures the analysis reflects completed surveys with self-reported gender identity. Missing variables were excluded from the analysis as follows: 11 patients did not report age, 23 did not specify glove use, 7 did not assess exam thoroughness, 1 did not state a preference, 3 did not indicate a preference for others’ glove use, 2 did not respond about disliking gloves, 4 did not answer about glove use at incision sites, and 6 did not comment on surgeon personability with glove use.

## Discussion

To our knowledge, this is the first study to assess patient perspective when comparing glove use during a physical examination by an orthopaedic surgeon in an outpatient setting. Historically, clinicians have been taught early on in medical training about proper glove use. Ultimately, gloves in the outpatient setting are often guided on the discretion of the surgeon. This study aimed to explore patient perceptions about glove use during a clinical physical examination. Our results demonstrated that there was no significant difference in those that identified as male or female patients when asked their preferences on their provider wearing gloves during their own physical examination and during the examinations of other patients; however, both did agree on glove use when examining a wound. In addition, patients reported that their orthopaedic surgeon’s decision to use gloves did not affect the patients’ feelings about examination thoroughness or making the encounter feel less personable.

We observed that patients’ feelings about non-sterile glove use in an outpatient setting is multi-factorial. Wilson et al in 2017 shared some similarities with our results in that 24% of their respondents reported a positive opinion of glove use while perceiving the purpose of infection prevention. 63% of respondents conveyed a negative opinion of glove use in the public questionnaire.^3^ The negative opinions were reported perceptions of overuse, not changing gloves between tasks, health care worker protection rather than patient protection, gloves being used in place of hand hygiene, and concern of latex exposure. In addition, 25 respondents reported on a recent experience in the health care setting where they felt gloves were inappropriately used. Although our survey did not ask questions specifically regarding infection prevention or risk, our survey highlighted that patients were agreeable to having a surgical wound examined with gloves. In 2018, Walaszek et al^4^ attempted to examine the differences in both patient and medical staff perception on glove use and hygiene practices by administering multicenter questionnaires involving 459 respondents with 37.7% patients from all hospital departments and 62.3% health care workers which included physicians, nurses, and medical students. Similar to our results, they found that age did not significantly affect patient opinions on glove use; however, there was a trend for the oldest patients appreciating glove use. Patients from rural areas and those with lower education levels also attributed greater value to glove use in preventing health care–associated infections. Health care workers with the least seniority and medical students were found to ascribe more importance to gloves.

While our study did not specifically examine the level of education among respondents, patients across all groups demonstrated the sentiment that gloves should be used during wound examinations, which is likely consistent with most provider’s previous training on the appropriateness of glove use. In all, Walaszek et al^4^ propose that better staff education is required, particularly of younger students and providers, to address the improper use or overuse of gloves in instances where glove use is unwarranted. While this may be the case, acknowledging patient’s perceptions on the appropriateness of glove use may be necessary to ensure that effective glove use strategies, and hand-hygiene practices, are better received by patients.

Although our research focus was on an outpatient setting our results shared similarities and dissimilarities with a 2009 study that surveyed patients in the inpatient setting and found that patients overall agreed with the use of gloves while health care workers provided care. They analyzed the patients’ country of origin and found a significant difference in attitude depending on where patients were from.^5^ Interestingly, our results showed that the older population showed no statistical difference in glove use preference, while their results showed that the older population appreciated glove use. The use of non-sterile gloves during physical examinations has been widely recommended as a standard practice to protect the examiner and examinee in settings where there is an increased risk of contact with bodily fluids, open wounds, and toxins.^6^ In 2020, Lindberg et al^7^ explored the idea that continued glove use can increase the risk of infection. The data collection included observations on internal medicine wards from nurses, nurse assistants, and physicians. The results showed that “A risk of organism transmission was observed not only within, but also between different ward zones.” Similarly, Loveday et al^8^ observed 163 episodes over 6 different wards and found that 42% of the observations included inappropriate glove use and “(37%) episodes of glove use there was a risk of cross-contamination, most (48%) being associated with failure to remove gloves or with performing hand hygiene after use.” Both research projects highlighted the use of non-sterile gloves, increasing possible infection.

More recently, as of 2024, there has been a push by the Central London Community Healthcare NHS Trust (CLCH) to reduce unnecessary use of non-sterile disposable gloves by way of the “Gloves Off” campaign. Not only has this initiative aimed to reduce the risk of potential cross-contamination when gloves are used for tasks that have a low risk of infection or exposure to bodily fluids, but it also attempts to address greenhouse effects and carbon emissions. The 6.5 million non-sterile gloves used annually by the CLCH produce approximately 338 000 kg-CO_2_-eq.^9^ University College London Hospitals has implemented measures to encourage providers to stop reaching for gloves out of habit when they are unnecessary and instead opt for hand-hygiene practices, which they view as being just as safe.^10^ Implementing similar initiatives and measures in outpatient settings such as the Foot and Ankle Clinic can tremendously impact on reducing waste and may potentially improve patient opinions regarding clinics that prioritize sustainability when warranted. Orthopaedic surgeons at the Foot and Ankle clinic would use approximately 15 600 gloves annually if medical examination gloves were used for every encounter. The weight in kilograms of an individual pair of nitrile gloves is approximately 5 g and a total of 78 000 g or 78 kg, annually at this clinic. Total carbon emissions equate to about 1170 kgCO_2_e. Given this information along with the data from our study, our approximate current annual use is 2964 gloves, 14 820 g or 14.8 kg with total CO_2_ emissions equating to 222 kgCO_2_e.^10^

Although our research did not explore cultural and regional differences among patients, current literature explores how these differences can influence patient preferences. In 2016, Merwe et al distributed 500 questionnaires at Bloemfontein National District Hospital in South Africa with 473 being returned. A total of 63 of the questionnaires were excluded, and the remaining 410 were included for analysis.^11^ The key themes of the study were patient preference on providers’ dress code, conduct, and resources used. It was concluded that 92.4% of participants preferred that their provider wear medical gloves during the examination portion of the visit. The participants included those that lived in South Africa and were able to understand English, Afrikaans, or Sesotho. Another piece of literature highlights how Mexicans, Mexican Americans, and White Americans display a difference in preference regarding patient autonomy or provider paternalism. Thompson et al focus on how Western culture values autonomy over both community and divinity, while other cultures may value community and divinity over autonomy. Data were gathered by asking participants a series of questions in a basic interview. Results showed a significant difference in “autonomy vs paternalism” in the group of White Americans vs Mexicans. Mexicans were more likely to prefer a paternalistic provider, while White Americans were more likely to prefer autonomy.^12^ Highlighting cultural and regional differences is essential to deliver patient-centered and culturally competent care, especially as the United States has the second-largest Mexican community representation after Mexico.^13^

Previous research around personal protective equipment (PPE) and patient’s experience during and after the COVID-19 pandemic raised the question on how experiences have changed. In 2022, Pathan et al surveyed admitted orthopaedic patients about their experience with staff wearing PPE. A total of 39 patients were included in the analysis, 31 (79.5%) answered “yes” to encountering doctors/nurses wearing PPE/face masks, 33 (84.6%) answered “yes” to thinking that wearing PPE was appropriate given the current situation of COVID-19, 29 (74.4%) answered “yes” to feeling that they were able to communicate in the same way when the team caring for them while wearing a mask, 4 (10.3%) answered “yes” to PPE affecting rapport with the doctor, and 34 (87.2%) answered “no” to not feeling scared when providers were wearing masks, and 17 (43.6%) answered “yes” to preferring doctors to wear a badge with the face visible on it. Similarly, our survey results share consensus on personal protective equipment, particularly gloves, not affecting rapport and personability with provider.^14^

Provider practices surrounding hand hygiene and glove use have been largely discussed in the literature; however, provider beliefs or attitude has minimal representation. Our study focuses on preference from a patient’s perspective, but on the contrary in 2024, Offermans et al^15^ explored attitudes and beliefs from the health care team’s perspective, particularly intensive care unit (ICU) nurses and assistants. A total of 11 ICU nurses and 2 assistants were recruited in this qualitative explorative study. Data collection happened from interviews that were recorded and transcribed. The results suggest that the overall theme showed a lack of ownership and that negative attitudes were found to be connected to the workers’ personal and organizational beliefs. It was found that these attitudes are challenging to change without significant groundwork. This raises the question if there is a difference in perspective between patients and providers as it relates to the use of medical examination gloves.^15^

In addition to analyzing patient perspectives around glove use, it is important to discuss glove use as it relates to tactile sensitivity and dexterity, especially in surgical practices.^16^ We did not investigate such factors; however, the literature supports that tasks such as feeling for pedal pulses at a foot and ankle clinic visit can be impacted with wearing gloves. In 2020, Moog et al discuss how glove use has been shown to diminish tactile sensitivity and dexterity. The study looked at 6 different types of glove and habits that included “double-gloved,” ‘oversized “undersized.” Two-point discrimination and Semmes-Weinstein monofilament testing were used in 27 non-surgeons.^16^ Results showed that a significant difference was found between those that were gloved vs bare hand, and different glove types. In addition, oversized gloves did show a significant difference in sensibility, while undersized gloves and double gloving did not.

This study has several limitations: (1) surveys are commonly known to have some response bias as the nature of the survey distribution may influence answers the nature of the survey distribution, (2) there is a small sample size, and it was completed at a single clinic site, which may mean the findings cannot be generalized to other patient populations, (3) there was some missing data from glove utilization, age, and gender identification, which may have the overall generalizability between these groups but was kept within reported data to serve as survey preference data, apart from the impact of glove utilization, (4) the non-randomization of the survey categories such as groups that used gloves or those that did not, and finally, (5) surveys have pre-defined answers it is possible that the patient population does not accurately reflect a larger population.

## Conclusion

When asked about preference for glove utilization during a foot and ankle appointment, generally all patients agreed that physicians should use gloves when examining wounds on the foot and ankle, although patients generally did not agree that glove use resulted in a less thorough examination. Importantly, glove use did not negatively impact a patient’s perception of their physician’s demeanor during any of the visit types.

Given the overall preference for physicians wearing gloves, especially during wound care, health care institutions should consider standardizing glove use during physical exams, particularly when examining wounds to align with patient preferences. Although our study did explore age and gender, future research would benefit from investigating a larger patient population to ensure a more comprehensive analysis on the missing variables in our data such as gender identity, cultural background, and health status. Future longitudinal studies should explore patient preferences on glove use over time or as politics change health care guidelines or policies. By incorporating these recommendations, health care providers can build stronger patient-provider relationships that focus on patient expectations and overall patient satisfaction.

## Data Availability

The data sets used and/or analyzed during the current study are available from the corresponding author on reasonable request.

## Appendix A

### Survey Questionnaire

1. My orthopaedic surgeon wore gloves today

 Yes No

2. Please select 1 reason below on why you are here today

 New patient Pre-operative visit Post-operative visit Long-term follow-up

3. I thought my orthopaedic surgeon did a thorough physical exam today

 Strongly Agree Agree Neutral Disagree Strongly Disagree

4. I would prefer if my orthopaedic surgeon wore gloves when assessing my foot and ankle

 Strongly Agree Agree No preference Disagree Strongly Disagree

5. I would prefer if my orthopaedic surgeon wore gloves when assessing other patients

 Strongly Agree Agree No preference Disagree Strongly Disagree

6. I would prefer if my orthopaedic surgeon did not wear gloves while examining my foot and ankle

 Strongly Agree Agree No preference Disagree Strongly Disagree

7. I feel that my orthopaedic surgeon is less personable when they wear gloves during their examination

 Strongly Agree Agree No preference Disagree Strongly Disagree

8. If I have a surgical incision or wound on my foot or ankle, I prefer that my surgeon wear gloves while examining me

 Strongly Agree Agree No preference Disagree Strongly Disagree
